# Editorial: Advanced interventions for self-regulation and neuroplasticity

**DOI:** 10.3389/fnhum.2026.1837168

**Published:** 2026-04-16

**Authors:** Naama Schwartz, Sophie Clarke, Elsa Florence Fouragnan

**Affiliations:** 1 Department of Psychology, Reichman University, Herzliya, Israel; 2 School of Psychology, Faculty of Health, University of Plymouth, Plymouth, United Kingdom

**Keywords:** emotion regulation, neuroplasticity, personalized intervention, self-regulation, Transcranial Ultrasound Stimulation (TUS)

Neuroplasticity—the brain's capacity to change in response to experience and perturbation—supports recovery after injury, the maintenance of cognitive function in aging, and adaptive regulation of emotion and sleep. At the same time, the translation of neuroplasticity research into clinical and scalable practice is evolving. Rather than relying on single, static interventions, the field increasingly tests combinatorial and personalized approaches that aim to (a) modulate neural and physiological state, and (b) convert that state into durable functional gains through training, feedback, and context-specific practice.

This Research Topic, *Advanced interventions for self-regulation and neuroplasticity*, brings together five contributions that illustrate this shift across neuromodulation, behavioral intervention, digital therapeutics, and molecular/epigenetic strategies. Read as a set, the papers suggest that neuroplasticity can be treated as a modifiable readiness state that may be supported—briefly or over longer windows—by complementary interventions.

A critical advancement in inducing this plastic state is the evolution of non-invasive brain stimulation (NIBS). While traditional methods like Transcranial Magnetic Stimulation (TMS) have revolutionized cortical modulation, they struggle to reach deep subcortical targets without affecting overlying tissue. In a Perspective article, Dunsford et al. introduce Transcranial Ultrasound Stimulation (TUS) as a precision tool capable of overcoming this depth limitation. The authors highlight the potential of TUS to target deep-brain structures essential for self-regulation, such as the thalamus and amygdala, which govern sleep and emotion but are largely inaccessible to standard NIBS. Crucially, they propose a framework where TUS functions not as a standalone cure, but as a “primer” that induces a transient state of neuroplasticity. This priming prepares neural circuits to be more receptive to subsequent behavioral interventions, such as cognitive restructuring or mindfulness training, thereby bridging the gap between direct physiological modulation and psychological self-regulation.

The concept of combining central modulation with peripheral behavioral training is developed further in the neurorehabilitation domain. Liu et al. review interventions for post-stroke movement disorders and argue for a “central–peripheral” synergistic model. This model integrates repetitive transcranial magnetic stimulation (rTMS) that can modulate cortical excitability and readiness for reorganization, with constraint-induced movement therapy (CIMT) which supplies intensive, functionally targeted practice to translate that readiness into usable motor gains. Importantly, the review treats recovery as bidirectional: neural state influences learning capacity, and task-based practice shapes how neural changes stabilize. This complements the design logic emphasized by Dunsford et al.: stimulation is not presented as sufficient on its own, but as potentially more useful when paired with structured behavioral engagement.

Where Liu et al. focus on pairing neuromodulation with rehabilitation training, Tian et al. synthesize evidence for mind–body exercise as a multi-component intervention in older adults with mild cognitive impairment (MCI). Their systematic review and meta-analysis covers practices such as Baduanjin, Tai Chi, and aerobic dance, and links behavioral outcomes (including global cognition) with measurable neuroplasticity involving significant increases in gray matter volume in the hippocampus and anterior cingulate cortex, regions critically vulnerable to neurodegeneration. One contribution of this review is conceptual: mind–body practices are difficult to classify as purely “top-down” or “bottom-up.” They combine movement, interoceptive attention, structured practice, and often social/contextual engagement, making them intrinsically bidirectional. The review's attention to modality differences also supports a pragmatic point that recurs throughout the Topic: intervention categories are broad, and outcomes may depend on the specific components and dosage of practice.

As interventions become more multifaceted, the challenge of inter-individual variability becomes a primary hurdle. Jiao addresses this by proposing a unified therapeutic paradigm for Digital Therapeutics (DTx). This Mini Review explores the integration of music therapy, brainwave entrainment (such as binaural beats), and multisensory stimulation. The author argues that the future of these non-pharmacological interventions lies in “closed-loop” systems governed by Artificial Intelligence. By utilizing real-time biofeedback (e.g., EEG or heart rate variability), AI algorithms can dynamically adjust therapeutic parameters—such as the frequency of auditory beats or the complexity of musical patterns—to match the user's instantaneous physiological state. This adaptive model aims to solve the “one-size-fits-all” problem, offering a scalable pathway to personalized mental health care that accounts for individual neural diversity.

Finally, the sustainability of these structural and functional changes relies on a supportive molecular environment. Jiao et al. provide a comprehensive review of biochemical modulators, specifically focusing on curcumin. The authors frame curcumin not simply as an antioxidant, but as a sophisticated epigenetic modulator capable of “reprogramming” the aging brain. This review suggests that biochemical interventions serves as the foundational “software update” for the genome, creating the necessary molecular conditions for neurogenesis and synaptic plasticity that other physical and technological interventions seek to exploit.

Together, healthy individuals, cognition and behavior emerge from dynamic interactions among molecular signaling pathways, synaptic mechanisms, large scale network organization, bodily physiology, environmental context, and lived experience. Neural systems operate across multiple spatial and temporal scales, continuously balancing stability and flexibility to support learning, adaptation, and performance. Because plastic change is distributed rather than localized, and context dependent rather than linear, interventions that target a single mechanism are unlikely to fully harness the brain's adaptive capacity. A combinatorial framework is therefore conceptually aligned with the multidimensional architecture of neuroplasticity, recognizing that meaningful enhancement or resilience depends on coordinated engagement across interacting biological and behavioral systems.

Ultimately, the modest but vital impact of this Research Topic lies in offering a grounded, multi-disciplinary framework that demonstrates how the brain's intrinsic capacity for self-regulation and recovery is most effectively supported when technological, physical, and biochemical strategies are thoughtfully integrated rather than applied in isolation.

